# Diosgenin enhances liposome-enabled nucleic acid delivery and CRISPR/Cas9-mediated gene editing by modulating endocytic pathways

**DOI:** 10.3389/fbioe.2022.1031049

**Published:** 2023-01-09

**Authors:** Brijesh Lohchania, Abisha Crystal Christopher, Porkizhi Arjunan, Gokulnath Mahalingam, Durga Kathirvelu, Aishwarya Prasannan, Vigneshwaran Venkatesan, Pankaj Taneja, Mohan Kumar KM, Saravanabhavan Thangavel, Srujan Marepally

**Affiliations:** ^1^ Centre for Stem Cell Research, Christian Medical College Campus, Vellore, India; ^2^ Sharda University, Greater Noida, India; ^3^ Thiruvalluvar University, Vellore, India; ^4^ Manipal Academy of Higher Education, Manipal University, Manipal, India

**Keywords:** CRISPR/Cas9, cationic lipid, diosgenin, transfections, genome editing

## Abstract

The CRISPR/Cas9 system holds great promise in treating genetic diseases, owing to its safe and precise genome editing. However, the major challenges to implementing the technology in clinics lie in transiently limiting the expression of genome editing factors and achieving therapeutically relevant frequencies with fidelity. Recent findings revealed that non-viral vectors could be a potential alternative delivery system to overcome these limitations. In our previous research, we demonstrated that liposomal formulations with amide linker-based cationic lipids and cholesterol were found to be effective in delivering a variety of nucleic acids. In the current study, we screened steroidal sapogenins as an alternative co-lipid to cholesterol in cationic liposomal formulations and found that liposomes with diosgenin (AD, Amide lipid: Diosgenin) further improved nucleic acid delivery efficacy, in particular, delivering Cas9 pDNA and mRNA for efficient genome editing at multiple loci, including AAVS1 and HBB, when compared to amide cholesterol. Mechanistic insights into the endocytosis of lipoplexes revealed that diosgenin facilitated the lipoplexes’ cholesterol-independent and clathrin-mediated endocytosis, which in turn leads to increased intracellular delivery. Our study identifies diosgenin-doped liposomes as an efficient tool to deliver CRISPR/Cas9 system.

## Introduction

Applications of CRISPR/Cas9-based genome editing platforms are rapidly increasing in both basic and clinical research. In the CRISPR/Cas9 system, the Cas9 protein creates double-stranded breaks (DSBs), and single-stranded guide RNA (sgRNA) helps the nuclease to perform gene editing with precision (Sahel et al., 2019). The DSBs can be explored to introduce or delete the desired base sequences, which hold promising potential in both basic and translational research (Leonova and Gainetdinov, 2020). Cas9 nuclease can be delivered in plasmid DNA or mRNA form that expresses the nuclease inside the cell, or in a native protein form as ribonucleoprotein (RNP) for gene editing (Duan et al., 2021). Three different forms of Cas9 delivery have their advantages and challenges. Among these three options, the delivery of Cas9 in pDNA form is primarily explored in basic research owing to its ease of application (Pattni et al., 2015; Fajrial et al., 2020). A wide variety of possible delivery methods have already been developed for *ex vivo* gene editing (Shim et al., 2017). However, their applications remain limited due to challenges in expanding the edited cells and exorbitant costs (Lino et al., 2018). Among viral vectors, adenoviral, adeno-associated viral (AAV), and lentiviral vectors are widely used for the delivery of Cas9 system-encoding cassettes to targeted cells (Xu et al., 2019; Bulcha et al., 2021). Although viral carriers are highly efficient, their potential to induce carcinogenesis and immunogenicity are the major concerns for their therapeutic applications (Hardee et al., 2017; Xu et al., 2019). Limited DNA packaging capacity is a major roadblock to the delivery of large-scale genome editing systems such as TALENS and CRISPR/Cas9 (Lino et al., 2018). More importantly, clinical-grade, large-scale vector production is a major technical hurdle in the application of viral delivery (Bulcha et al., 2021). Non-viral delivery systems, in particular, lipid-based systems were found to be promising alternatives owing to their robustness in manufacturing and ease in their applications (Rajesh et al., 2007; Kulkarni et al., 2018; Wei et al., 2020). Non-viral systems offer advantages over viral vectors with low immunogenicity and no insertional mutagenesis, which could minimize the chance of short-term and long-term adverse effects (Check, 2003; Prata et al., 2004; Kay, 2011; Yin et al., 2014). Also, their ability to deliver larger genetic payloads makes cationic lipids an attractive alternative for the CRISPR/Cas9 delivery system (Wang et al., 2020). More importantly, recent advances in the past few years in exploring mRNA technology for *in vivo* gene editing paved the way for a novel class of gene-editing therapeutics. Gillmore et al. successfully edited the transthyretin (TTR) gene in the adult human liver for treating human hereditary transthyretin (hATTR) amyloidosis by intravenously injecting lipid nanoparticles encapsulating Cas9 mRNA and TTR gene targeting single guide RNA (sgRNA) (Batista and Flotte, 2021; Gillmore et al., 2021). Inspired by the success of lipid nanoparticle-mediated mRNA vaccines for SARS-CoV2 and *in vivo* gene editing, several attempts have been made to develop a suitable delivery vector for multiple therapeutic applications.

In our previous efforts to develop efficient nucleic acid delivery lipid systems, we evaluated the effect of the molecular architecture of the lipid, including the hydrophilic head group, hydrophobic variations, and linker functionalities (Mukthavaram et al., 2009). From the structure-activity investigations, liposomes of amide linker-based cationic lipids were found to be effective in delivering various nucleic acids, including pDNA, siRNA, shRNA, and microRNA. However, the transfection efficiencies of these liposomes were found to be lower with larger plasmids (greater than 10 kb) such as CRISPR/Cas9 plasmids. It is a well-established phenomenon that the transfection efficiencies of plasmid DNA also depend on its size. Smaller plasmids can be easily delivered, whereas delivery of larger plasmids is a challenge (Mahalingam et al., 2022b). To improve the transfection efficiencies of the cationic lipids, we started exploring the other constituents of the lipid nanoparticle system as alternatives to cholesterol. In our prior findings, we demonstrated that using steroidal spirosolane and tomatidine as an alternative to cholesterol in the liposomal formulations would enhance nucleic acid transfections by permeabilizing the membrane (Rangasami et al., 2019). Marie C.J. et al. Demonstrated that sapogenins could be used to permeabilize the cell membrane by solubilizing the cholesterol without affecting its structure for delivering antigens (Jacob et al., 1991). However, there are no reports exploring their role in nucleic acid delivery. Taking cues from previous findings, including our own, in the present study we chose the aglycone portion of steroidal sapogenins. The primary difference is that cholesterol has a heptyl chain tethered to the steroidal portion, whereas the spiroacetal ring in place of the alkyl chain is present in sapogenins. Owing to the surfactant nature of the sapogenins, they can interact with the plasma membrane and may offer help in facilitating the larger nucleic acids. To better understand the influence of sapogenins on liposomal transfections, we prepared liposomes with different sapogenins as a co-lipid with our previously identified transfection-efficient amide lipid to further enhance intracellular delivery of nucleic acids and gene-editing efficiencies.

## Materials and methods

**Isolation of plasmid:** The eGFP plasmid (Green Fluorescent Protein encoding reporter gene) was amplified in the DH5α-strain of *Escherichia coli.* The plasmid was isolated using the MaxiPrep method with alkaline lysis buffer, and the purity was analyzed by the A_260_/A_280_ ratio (∼1.9) using NanoDrop 2000. 1.5% agarose gel electrophoresis was performed to check the same.

**Preparation of liposomes:** 1 mM liposomes were prepared using previously established protocols (Chandrashekhar et al., 2011). In brief, the relevant amounts of lipids were dissolved in chloroform in a glass vial and formed a thin film. They were dried for 8 h, hydrated overnight, and probe-sonicated to form SUVs (Table 1).

**TABLE 1:** Liposome compositions.

S. No.	Cationic lipid	Concentration (mM)	Co-lipid	Concentration (mM)	Liposome
1	Amide lipid	1	Cholesterol	1	AC
2	Amide lipid	1	Diosgenin	1	AD
3	Amide lipid	1	Smilagenin	1	ASm
4	Amide lipid	1	Sarsapogenin	1	ASa
5	Amide lipid	1	Tigogenin	1	AT
6	Amide lipid	1	Yamogenin	1	AY

**Zeta potential (ξ) and size measurements:** Liposome sizes and surface zeta potentials were measured on a Lite Sizer™500 Particle Analyzer (Anton Paar, Austria). The diameter of the particles was analyzed in Milli-Q DI water using an established protocol (Maddila et al., 2021).

**IVT mRNA synthesis:** The chemically base-modified eGFP and Cas9 mRNAs were synthesized by an optimized enzymatic *in vitro* transcription (IVT) protocol as described previously (Mahalingam et al., 2022a). Briefly, eGFP and Cas9 IVT templates were prepared by PCR using T7 promoter-containing primers. The chemically modified mRNAs were transcribed from purified IVT templates by replacing UTP with methyl pseudouridine 5′-triphosphate (me1Ψ-UTP) in the T7 polymerase reaction mix and purifying transcribed RNA by ammonium acetate precipitation. Later, Cap one was added at the 5′ ends and a poly-A tail around 150 base lengths at the 3′ end of modified RNA using enzymatic, capping, and poly-A tailing methods. The chemically modified mRNAs were purified and used for transfection experiments. The purification of modified mRNA was done by organic extraction and ammonium acetate precipitation. Briefly modified mRNAs were separated from the IVT reaction mix by TE-saturated phenol/chloroform at pH 8.0. The mRNA-containing aqueous upper phase was precipitated with 5 M ammonium acetate after centrifugation, and the precipitated mRNA was pelleted by centrifugation at 12,000 *g* for 15 min at 4°C, followed by washing with 70% ethanol and re-suspending the pellet in RNase-free water. The purified mRNA was stored at -80 or used for further studies.

**Transfection assay:** HEK 293 T cells were seeded at a density of 50,000 cells per well in a 24-well plate 12–18 h before the transfection. 0.5 μg of eGFP plasmid DNA was complexed with different liposomes in serum-free media (total volume made up to 100 μL) for 30 min. The lipid has base (+/-) charge ratios of 1:1. The complexes were then added to the cells. The transfection profiles obtained on different days were identical. In serum-free media (total volume made up to 100 μL) for 30 min, 0.2 μg of eGFP mRNA was complexed with different liposomes for mRNA transfections. The lipid has base (+/-) charge ratios of 2:1. The complexes were then added to the cells.

**Endocytosis blocking:** Endosomal blocker studies were performed using established protocols (Dharmalingam et al., 2017). In a 24-well plate, 50,000 HEK293 T cells were seeded and cultured at 37°C and 5% CO_2_ for 24 h. 30 min before the addition of the lipoplexes, cells were pretreated with sucrose (450 nM), methyl-β-cyclodextrin (2 mM), dynasore (80 μM), chlorpromazine (15 μM), and nystatin (80 μM) in plain DMEM individually. The medium was replaced with DMEM containing 10% FBS and 1% penicillin−streptomycin. The lipoplexes were added to the cells, and the cells were incubated at 37°C and 5% CO_2_. The reporter gene activity was quantified after 48 h using flow cytometric analysis.

**Cholesterol trafficking experiment:** HEK293T cells were seeded in a 24-well plate at 50,000 cells per well. After 4 h, imipramine was added in different concentrations of 10, 20, and 40 μM and allowed to grow for 24 h. Then the cells were treated with AC 1:1 and AD 1:1 lipoplexes with a 4:1 lipid: DNA charge ratio. After 48 h, cells were imaged under the epifluorescence microscope.

**CRISPR/Cas9 pDNA transfection:** HEK293T cells were seeded at a density of 50,000 cells per well in a 24-well plate 12–18 h before transfection. 0.5 μg of 4.7 kb eGFP-N1, 11.7 kb pL-CRISPR-EFS-GFP, and 13.7 kb pL-CRISPR-SFFV-GFP (pL-CRISPR-EFS-GFP and pL-CRISPR. SFFV.GFP were a gift from Benjamin Ebert - Addgene plasmid #57827) [18], and the lentiCRISPR-AAVS1 sgRNA construct (with lentiCRISPR-AAVS1 sgRNA a gift from David Sabatini - Addgene plasmid #70661) (19) was condensed with P3000 DNA-condensing agent and then formed a complex with AD, AC and LF3000 liposomes respectively in plain DMEM/MEM medium (total volume was 100 μL) for 30 min. The lipid:DNA (+/-) charge ratio was 1:1 for both AD and AC and 1 μL of LF3000 for each well of the 24-well plate, as stipulated. The complexes were then added to the cells. The transfection profiles obtained on different days were identical.

**Cas9 mRNA Transfection:** In the HEK 293 cell line, Cas9 mRNA and sgRNA targeting the SCD locus of β-globin were transfected using AD, AC, and commercial control. 200 ng of cas9 mRNA were used in the lipid:mRNA (+/-) ratio of about 2:1 and incubated for 15 min at room temperature. The mixture was added to the cells.

**Generation of cas9 expressing 293-T cell lines:** HEK293 T cells (4 × 10^5^) were cultured in six-well cell culture plates (Corning). When cells reached 80% confluence, 2.5 μg of the lentiCRISPR V2 (Addgene #52961) plasmid was transfected using Lipofectamine™ 3,000 Reagent as per the manufacturer’s protocol. 72 h post-transfection, 2.5 μg/ml of puromycin was added to the transfected cells for antibiotic selection. After selection, the cell line was maintained with 1 μg/ml of puromycin to obtain a stable HEK 293-T Cas9 cell line.

**T7 Endonuclease I (T7E1) Assay:** 48 h post-transfection, the cells were pelleted at 2000 rpm for 5 min. 100 µL of QE DNA Extraction Buffer was added to the pellet, which was incubated at 65°C for 30 min and 98°C for 15 min. DNA was quantified by the iQUANT nanodrop. To detect the editing, the AAVS1 region was amplified using the primers 5′Forward- TTCGGGTCACCTCTCACT and 3′Reverse- GGC​TCC​ATC​GTA​AGC​AAA​CC, and the amplicon size was 469 bp. Similarly, we used the Forward and Reverse primers TAG​CAA​TTT​GTA​CTG​ATG​GTA​TGG and 3′Reverse- GGA​AAA​TAG​ACC​AAT​AGG​CAG​AGA for the β-globin locus, and the amplicon size was 496 bp. Samples were run on 2% Agarose Gel with a 100 bp ladder to confirm the amplicon size. After amplifying the respected target region, the PCR product was denatured and re-annealed to allow heteroduplex formation. The samples were treated with 10 units of T7 endonuclease I enzyme for 20 µL of hetero-duplex samples, and after digestion, the resulting cleaved bands were visualized by PAGE. The indel percentage was measured using ImageJ software.

**Statistical analysis:** The experimental data in each graph are represented as the mean ± SD of obtained values from each experiment, performed at least three times. Experimental data from each group were compared with other groups within the graph using the student t-test. A *p* < 0.05 was considered significant.

## Results

### Screening of sapogenins as co-lipids

**FIGURE 1:**
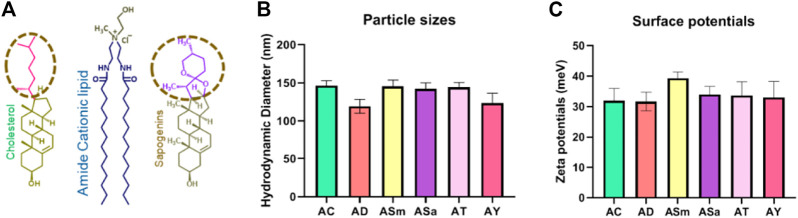
Liposomal physicochemical characterizations. Schematic representation of the lipids used in the liposomal formulations **(A)**, Hydrodynamic diameters of liposomes **(B)**, and Surface potentials of the liposomes **(C)**.

To understand the effect of incorporating steroidal sapogenins into the liposomes, firstly, we characterized the physicochemical properties, including particle sizes, surface potentials, and DNA-binding properties. Hydrodynamic diameters of Liposomes of AC (Amide: Cholesterol), ASm (Amide: Smilagenin), and ASa (Amide: Sarsapogenin) were found to be similar within the range of 150 nm ± 11, whereas slightly lesser for AD (Amide: Diosgenin) and AY (Amide: Yamogenin) found to be around 120 nm ± 8 in dynamic light scattering (DLS) studies (Figure 1B). The surface potentials of all the liposomes were found to be positive in the range of +25 to +40 meV (Figure 1C). DNA binding properties of all the liposomes demonstrated strong binding even at a 1:1 lipid:DNA charge ratio (Supplementary Figure S1). Further, we evaluated the strength of DNA binding with an anionic polymer, heparin with a heparin displacement assay (Supplementary Figure S2), and protection from DNase with a DNase-I sensitivity assay (Supplementary Figure S3). Both experiments confirmed that liposomes protect DNA from external influences. Overall, there is no significant difference in the physicochemical properties of liposomes either with cholesterol or other sapogenins (Figure 1).

### *In vitro* nucleic acid transfections

**FIGURE 2:**
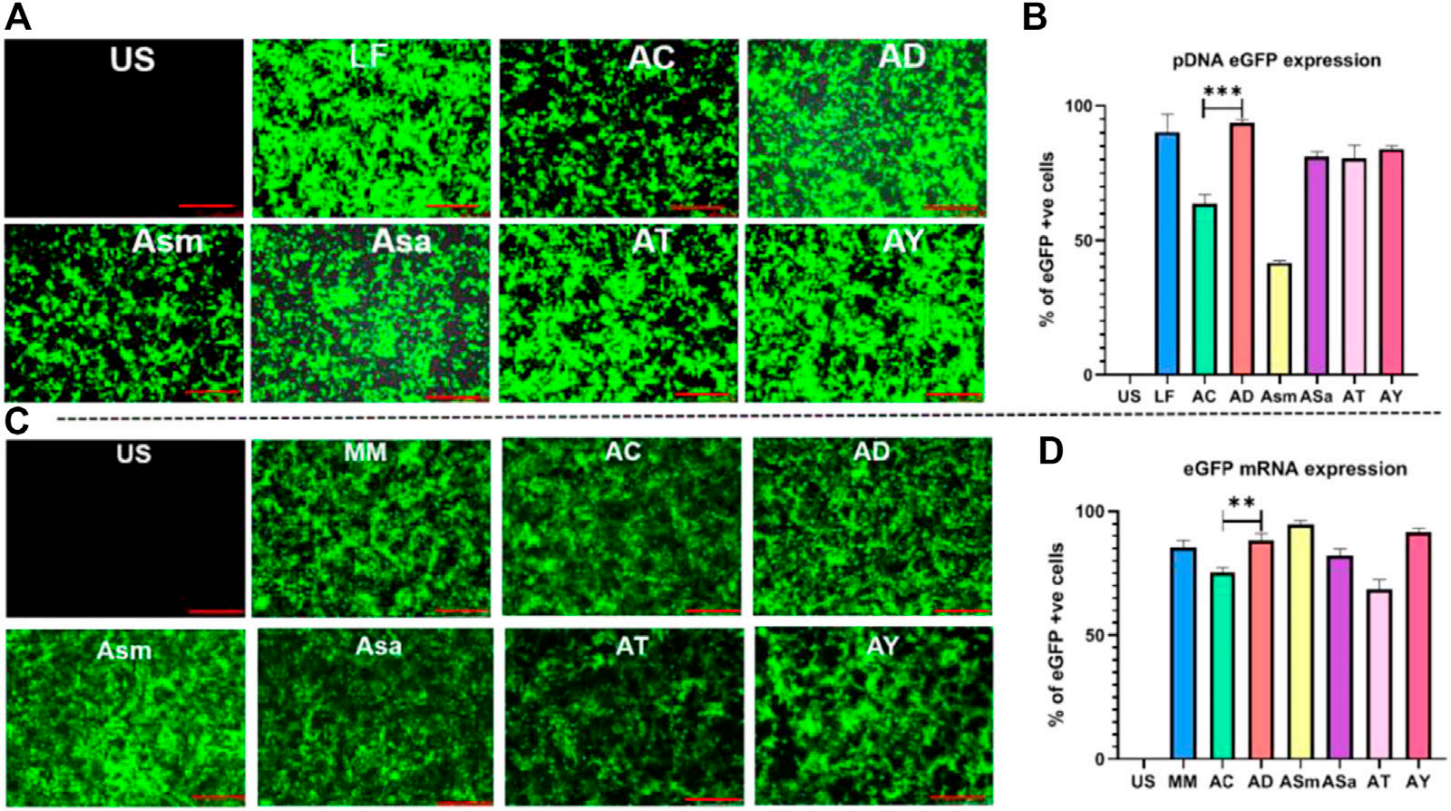
Transfection studies with pDNA and mRNA of different liposomes. Screening of sapogenin liposomes using the eGFP pDNA reporter gene, Representative images in epifluorescence microscope **(A)**, Flow cytometry analysis of eGFP pDNA expression **(B)**, Screening of sapogenin liposomes using eGFP mRNA, Representative images in epifluorescence microscope **(C)**, Flow cytometry analysis of eGFP mRNA expression **(D)**.

Firstly, we evaluated the gene delivery efficiencies of the liposomes in representative HEK293T (human embryonic kidney cells) at a lipid/DNA charge ratio of 1:1 using eGFP N1 plasmid DNA as the reporter gene encoding the Green Fluorescent Protein, as described previously (Dharmalingam et al., 2017). AD, ASa, AT, and AY showed an almost similar percentage of GFP-positive cells around 85–90%, and showed 20–30% superior transfections when compared to AC liposomes (Figure 2A,B). Transfection efficiencies of AD liposomes were found to be as efficient as those of the commercial transfection reagent, Lipofectamine 3000 (Figure 2B).

Next, we evaluated the transfection efficiencies of these liposomes in delivering linear nucleic acids and mRNA in the HEK-293 T using GFP-encoding mRNA (Figure 2C,D). Among the sapogenin-doped liposomes, AD, ASm, and AY liposomes showed around 20% superior transfections compared to AC liposomes. Interestingly, ASm liposomes showed 2-fold-increased mRNA transfection properties compared to pDNA transfection (Figure 2D). Transfection efficiencies of AD liposomes were found to be as efficient as those of commercial transfection reagent, LF messenger Max (Figure 2D). We have also compared the transfection efficiencies of AC and AD liposomes in CHO cells. Similar trends were observed in HEK293T cells (Supplementary Figure S4). AD liposomes demonstrated superior mRNA transfection over AC liposomes (Supplementary Figure S4). Overall, steroidal sapogenins, particularly diosgenin-doped AD liposomes, were found to be efficient in delivering nucleic acids, both circular pDNA, and linear mRNA, as efficiently as commercial controls. Considering the wider availability and lower cost, we proceeded further with diosgenin.

### Evaluating the lipoplex endocytosis

**FIGURE 3:**
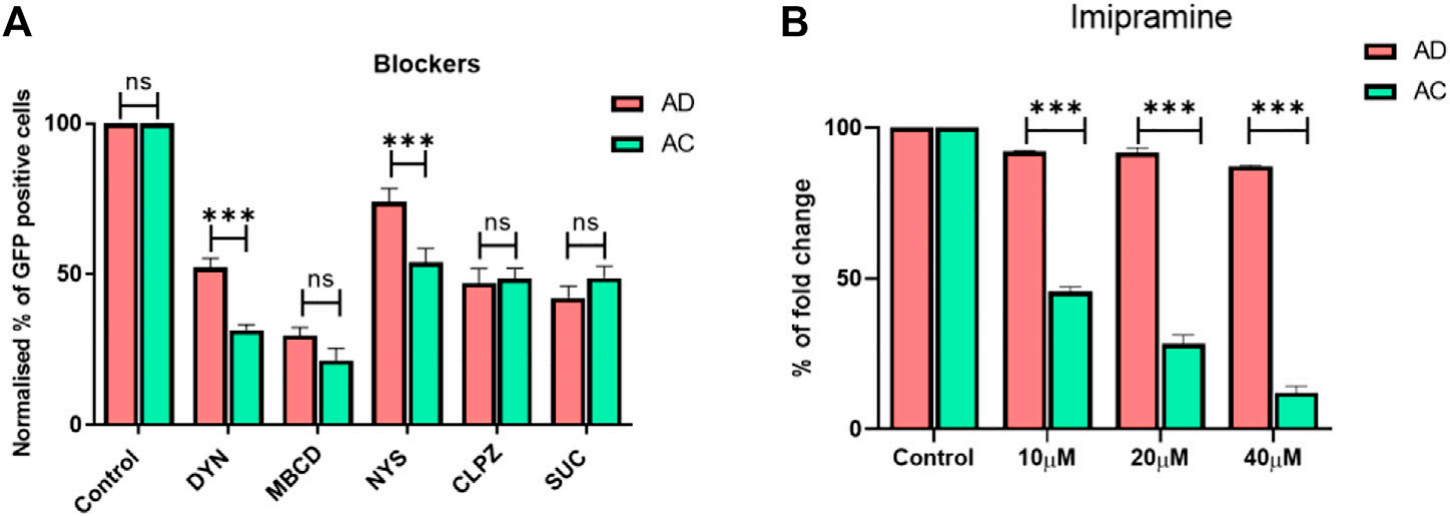
Transfections in the presence of endocytosis blockers. Normalized eGFP expression by endocytosis inhibitors. HEK-293 cells were pretreated with Dynosore (80 μM), M-β-CD (2 mM), Nystatin (80 μM), Chlorpromazine (15 μM), and Sucrose (450 mM) in serum-free media for 1 hour before exposure to the 1:1 lipid/DNA charge ratio of AC and AD lipoplexes, respectively **(A)** Imipramine was added in the different concentrations of 10, 20, and 40 μM after 4 h of seeding of HEK293 cells, and then the cells were treated with AC and AD lipoplexes with a 1:1 lipid-to-DNA charge ratio (*n* = 3; **p < 0.001 compared with AC and LF3000) **(B)**.

To probe the influence of diosgenin on endosomal pathways of AD lipoplexes for intracellular delivery, we treated the cells with AD lipoplexes and compared them with cholesterol-doped AC lipoplexes (Figure 3A), in the presence of endocytosis inhibitors. Chlorpromazine, a clathrin-mediated endocytosis inhibitor reduced transfection by 50% in both AC and AD lipoplexes (Figure 3A). Caveolae blocker nystatin reduced 50% of transfections of AC lipoplexes, whereas only 30% of transfections were affected with AD lipoplexes. Methyl-β-cyclodextrin (M-β-CD), a cholesterol-depleting agent (Ilangumaran and Hoessli, 1998), reduced the transfection to around 25% in both lipoplexes. (Figure 3A). With Dynosore, a dynamin-dependent pathway blocker, transfections of AC lipoplexes were reduced by about 70%, whereas 50% of the transfections were reduced in AD lipoplexes. Overall, transfections with endocytic blockers revealed that endocytosis of AD lipoplexes was less dependent on caveolae-mediated and dynamin compared to AC lipoplexes. AD lipoplexes exhibited more cholesterol-independent internalization than AC lipoplexes.

### Evaluating cholesterol dependence on endocytosis of the lipoplexes

To further understand the influence of plasma membrane cholesterol on endocytosis of the lipoplexes, cells were pre-treated with imipramine, which inhibits both the synthesis of cholesterol and trafficking from late endosomes and lysosomes to the plasma membrane and endoplasmic reticulum. With an increase in the concentration of imipramine, transfection efficiencies of AC lipoplexes were reduced in an inversely proportional manner; at a 40 µM concentration, the transfection efficiencies of AC lipoplexes were reduced to <10%, whereas AD liposomes manifested no significant effect on transfection efficiencies even in the presence of 40 µM imipramine. Our results support the hypothesis that AD endocytosis and intracellular transport are less dependent on cholesterol trafficking. (Figure 3B).

### Evaluating the different sizes of CRISPR/Cas9 plasmid transfections

**FIGURE 4:**
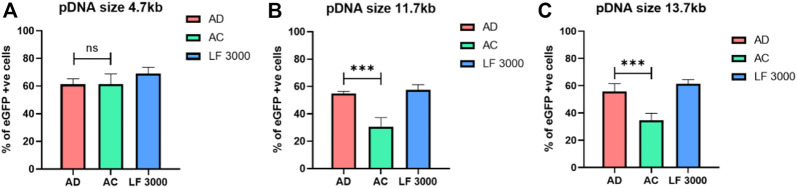
Delivery of varied sizes of plasmids. AD, AC, and LF3000 liposomes delivered a 4.7 kb eGFP-N1 plasmid **(A)** and larger size plasmids, i.e., 11.7 kb pL-CRISPR. EFS.GFP **(B)** and 13.7 kb pL-CRISPR. SFFV.GFP plasmid construct respectively (compacted with P3000 DNA-condensing agent) **(C)** (*n* = 3; **p < 0.001 compared with AC and LF3000).

To explore the efficiency of AD liposomes in delivering the genome-editing system, we evaluated its efficiency with the 11.7 kb pL-CRISPR. EFS.GFP (Addgene #57818) and 13.7 kb pL-CRISPR. SFFV.GFP (Addgene #57827) plasmid constructs, which express both Cas9 and eGFP proteins driven through the EFS or SFFV promoter as a single transcript. (Heckl et al., 2014). The amount of Cas9 protein expression is directly correlated with the amount of eGFP expression, as the single transcript is producing both Cas9 and eGFP proteins, aided by the self-cleaving P2A peptides, which work through ribosomal skipping of peptide bond formation at the terminal proline residue in the P2A peptide, resulting in the production of individual polypeptides. AD liposomes delivered 11.7 kb and 13.7 kb CRISPR-GFP plasmids ∼80% and ∼60% more effectively than AC liposomes, respectively (Figure 4B,C). AD liposomes could deliver larger plasmids as efficiently as the commercial control LF3000. Further studies directed toward the combined use of AD liposomes with DNA-condensing agents for delivering larger-size plasmids such as CRISPR plasmids are warranted. AD liposomes exhibited successful delivery of a wide range of nucleic acids from 1 to 13 kb in linear and circular constructs.

**FIGURE 5:**
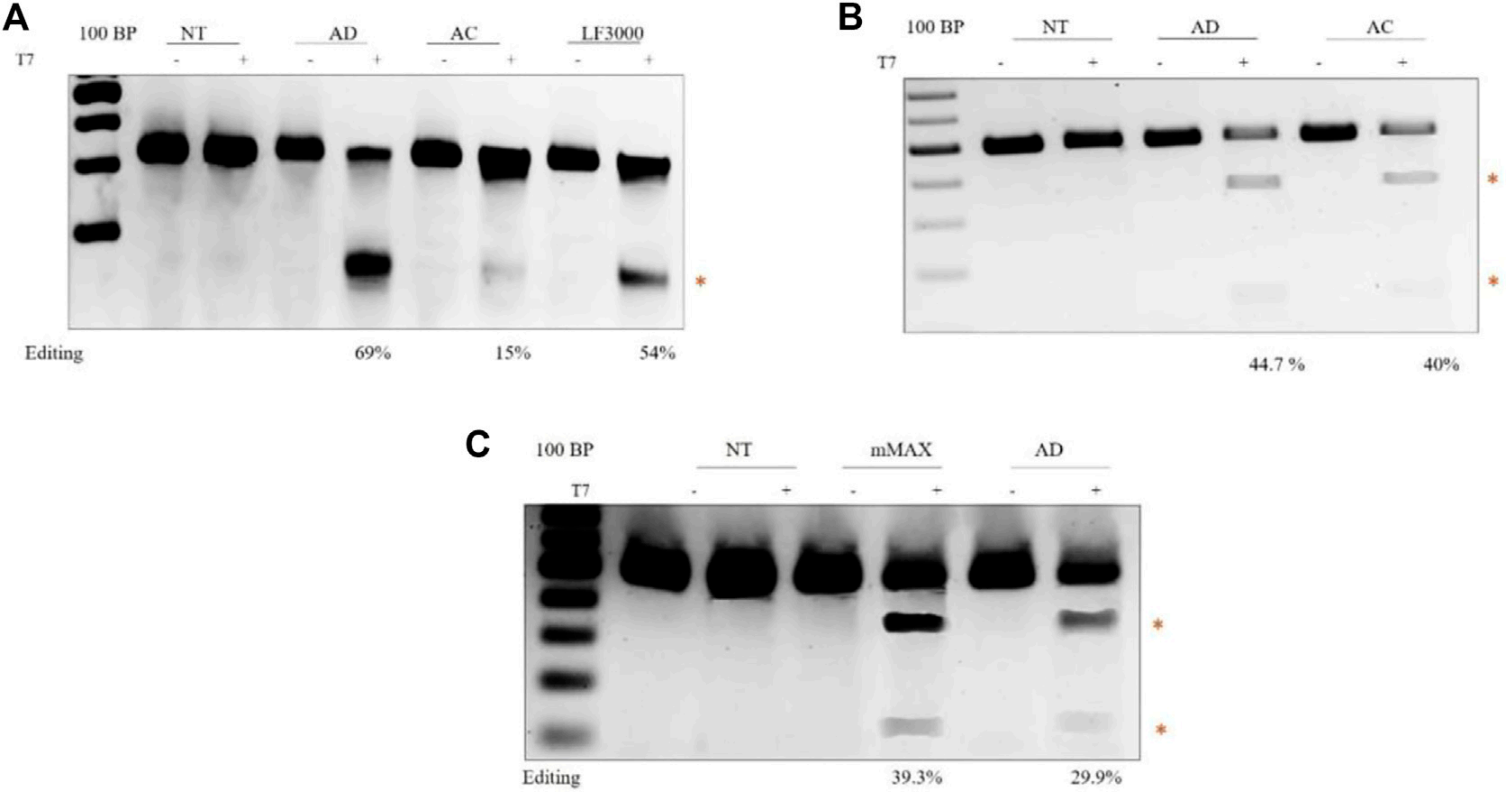
Delivery of different forms of CRISPR/Cas9 in HEK-293T cell lines: Agarose gel electrophoresis showing T7 activity. The cleaved product is highlighted in red. The editing efficiencies are indicated at the bottom of the gel. CRISPR-Cas9 plasmid DNA-mediated gene editing. Transfection of lenti-plasmid CRISPR-AAVS1 sgRNA, which expresses human codon-optimized Cas9 protein and an AAVS1-targeting sgRNA element from the U6 promoter, using transfection reagents AD, AC, and LF3000 **(A)**. sgRNA mediated gene editing in Cas9-expressing cells. sgRNA targeting the SCD locus was transfected with AD and AC **(B)**. Cas9 mRNA mediated gene editing. Transfection of SCD sgRNA and Cas9 mRNA targeting the Hbb locus using Mmax, AC **(C)**.

**CRISPR/Cas9 genome editing.** To directly analyze the efficiency of diosgenin-doped liposomes in mediating CRISPR/Cas9 genome editing, we transfected HEK293 T cell lines with a 14 kb All-in-One CRISPR plasmid, which expresses both Cas9 and sgRNA. The sgRNA targets the AAVS1 locus. AD liposomes showed superior indel efficiency of 69%, compared to AC liposomes of 15%, and comparable with commercially available Lipofectamine^®^ 3,000s 54% success rate in cleaving the AAVS1 locus (Figure 5A). These results are in concurrence with the larger CRISPR pDNA transfections. Editing experiments with Cas9 delivery in pDNA convincingly demonstrated that using Diosgenin as a co-lipid in AD liposomes increased editing efficiency increased by two to three folds compared to cholesterol-doped AC liposomes.

There is a growing interest in delivering Cas9 in mRNA form for *in vivo* gene editing. Multiple efforts in pre-clinical models successfully demonstrated that delivery of mRNA in Cas9 form with lipid nanoparticles enabled *in vivo* gene editing (Tabebordbar et al., 2016; Luther et al., 2018). Recently, the delivery of Cas9 mRNA in lipid nanoparticles showed successful phenotypic correction in transthyretin amyloidosis (hATTR) (Tabebordbar et al., 2016). To this end, we first evaluated the efficiency of AD liposomes in delivering chemically synthesized sgRNA. The HEK293 T cells stably expressing Cas9 were transfected with the sgRNA targeting the sickle cell disease locus. The T7 endonuclease assay showed higher levels of AD liposome-mediated gene editing in 44.7% of cells than AC liposomes, which mediated sickle cell locus editing in 40% of cells (Figure 4B). Since sgRNAs are small, around 120 bases, there is no notable difference in the editing efficiencies between AD and AC liposomes. Next, we combined Cas9 mRNA and sgRNA targeting the sickle cell disease locus. When compared to commercial messenger Max, the percentage of indels was somewhat lower in AD liposomes, around 40% vs 30% in delivering mRNA (Figure 4C). However, considering mRNA transfections and the ability of AD liposomes to protect from enzymatic degradation, AD liposomes may be a potential carrier system for *in vivo* editing. Overall, our studies demonstrated that AD liposomes were found to be a potential delivery system for Cas9 in plasmid DNA and mRNA forms.

## Discussion

In this study, to deliver larger plasmids, including CRISPR plasmids, effectively into the cells, we screened functionally active steroidal sapogenins as an alternative co-lipid to cholesterol. Apart from serving similar functions as cholesterol in providing physical stability to the liposomes, it additionally permeabilizes the membrane by solubilizing the cholesterol present in the plasma membrane without affecting its integrity (Jacob et al., 1991). Hence, we chose to screen steroidal sapogenin to improve nucleic acid transfections of the cationic liposomes. Sapogenins are analogous to cholesterol with a spiro-acetal ring attached to their steroidal moieties (Parama et al., 2020). From the screen of sapogenins, we demonstrated that cationic liposomes comprising diosgenin exhibited enhanced transfection efficiencies in our previous liposomal formulations of amide cholesterol (AC). Diosgenin and yamogenin-doped liposomes have particle sizes of around 120 nm. This could be due to the surfactant properties that influence the liposome’s intra-lipid arrangement. The DNA binding properties of sapogenin-doped liposomes were found to be similar to those of cholesterol-doped liposomes, which indicates that the sapogenins are not affecting the nucleic acid binding properties. Among the other sapogenins, diosgenin was found to be superior in imparting nucleic acid delivery properties to liposomes. Sapogenin-doped AD liposomes could deliver both larger circular plasmid DNAs and linear nucleic acid molecules (mRNA) than AC liposomes. The superior intracellular delivery of AD liposomes could be attributed to their increased cholesterol-independent internalization. This could be the reason for the superior transfections of AD liposomes, as clathrin-mediated endocytosis is preferred over clathrin-independent endocytosis (CIE). We further demonstrated that the liposomes from AD liposomes effectively delivered genome-editing tools containing CRISPR/Cas9-encoded pDNA, mRNA and had an enhanced genome-editing efficiency compared to our previously reported AC liposomes. More importantly, we could demonstrate that by modulating the endocytic pathways of the lipoplexes by employing steroidal sapogenin in the cationic liposomes, the intracellular delivery of multiple biomolecules, including nucleic acids and proteins, could be enhanced. Overall, single transfection reagent AD liposomes were found to be as efficient in delivering plasmid DNAs as Lipofectamine 3,000 and Lipofectamine Messenger Max in delivering mRNAs.

## Conclusion

In summary, our findings collectively demonstrate that diosgenin can be used as a co-lipid to improve nucleic acid transfections and the gene editing efficiency of cationic liposomes. This provides an efficient alternative to cholesterol in developing lipid nanoparticle formulations for multiple nucleic acid delivery applications, including nucleic acid delivery and gene editing.

## Data Availability

The original contributions presented in the study are included in the article/Supplementary Material, further inquiries can be directed to the corresponding authors.
