# Supplementary material for: Diosgenin enhances liposome-enabled nucleic acid delivery and CRISPR/Cas9-mediated gene editing by modulating endocytic pathways
Source: Front Bioeng Biotechnol. 2023 Jan 9;10:1031049. doi: 10.3389/fbioe.2022.1031049 (PMC9868636; doi:10.3389/fbioe.2022.1031049)
Supplement: Supplementary file 1 [file DataSheet1.PDF]

Diosgenin enhances liposome-enabled nucleic acid delivery and CRISPR/Cas9 mediated gene editing by modulating endocytic pathways

Brijesh Lohchania[1,2#], Abisha Crystal Christopher[1,3#], Porkizhi Arjunan[1,4], Gokulnath Mahalingam[1], Durga Kathirvelu[1], Aishwarya Prasannan[1], Vigneshwaran Venkatesan[1], Pankaj Taneja[2], Mohan Kumar KM[1], Saravanabhavan Thangavel[1*] and Srujan Marepally[1*]

[1] *Centre for Stem Cell Research, Christian Medical College Campus, Bagayam, Vellore 632002, India.*

2 Sharda University, Greater Noida, Uttar Pradesh, 201310, India

[3] *Thiruvalluvar University, Tamil Nadu, India;*

[4] *Manipal Academy of Higher Education, 76793, Manipal, Karnataka, India;*

# *Equally contributing first authors*

*** Corresponding authors:**
srujankm@cmcvellore.ac.in;
sthangavel@instem.res.in;

1. **Gel Retardation – pDNA**

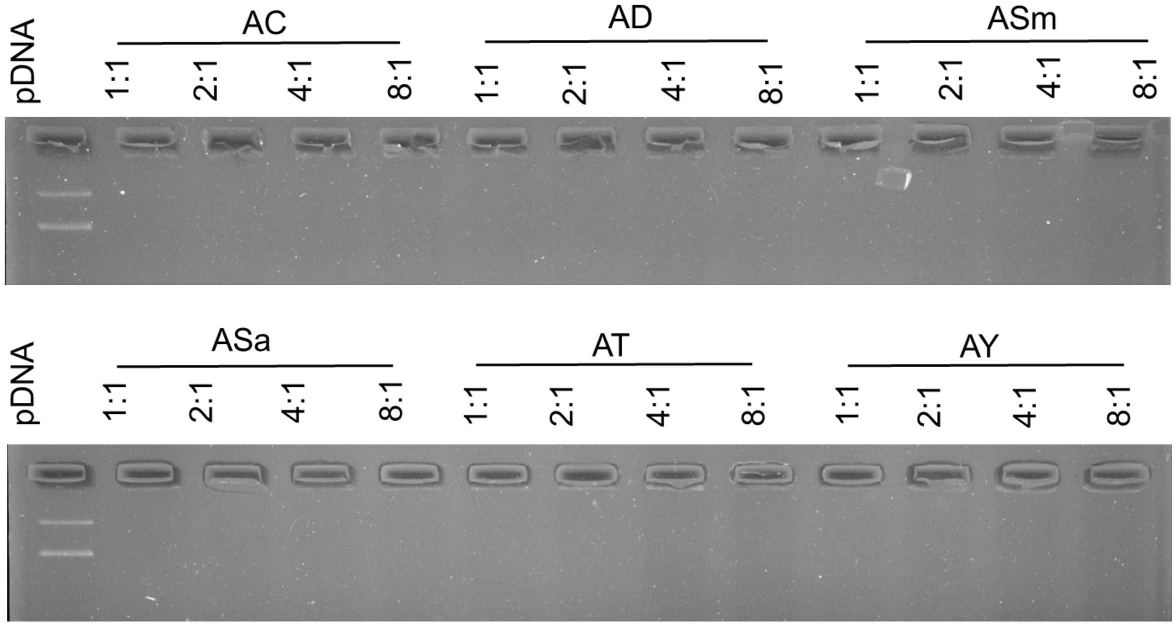

Figure S1: Gel Retardation Assay with pDNA of different sapogenin liposomes in varying charge ratios. The absence of bands indicates the complete encapsulation of pDNA by liposomes in all the ratios.

2. **Heparin Displacement assay**

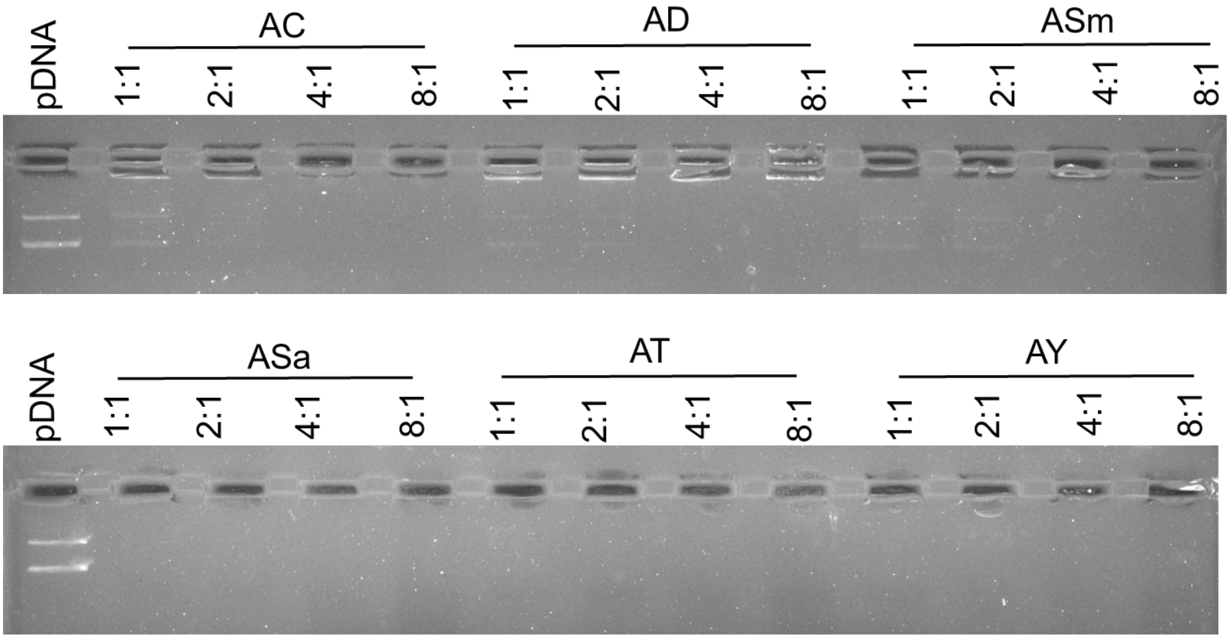

Figure S2: Heparin Displacement Assay with pDNA of different sapogenin liposomes in varying charge ratios. The liposomes encapsulated the nucleic acid even in the presence of another negatively charged molecule, Heparin (0.5µg) is shown by the absence of bands.

3. DNase I Sensitivity Assay

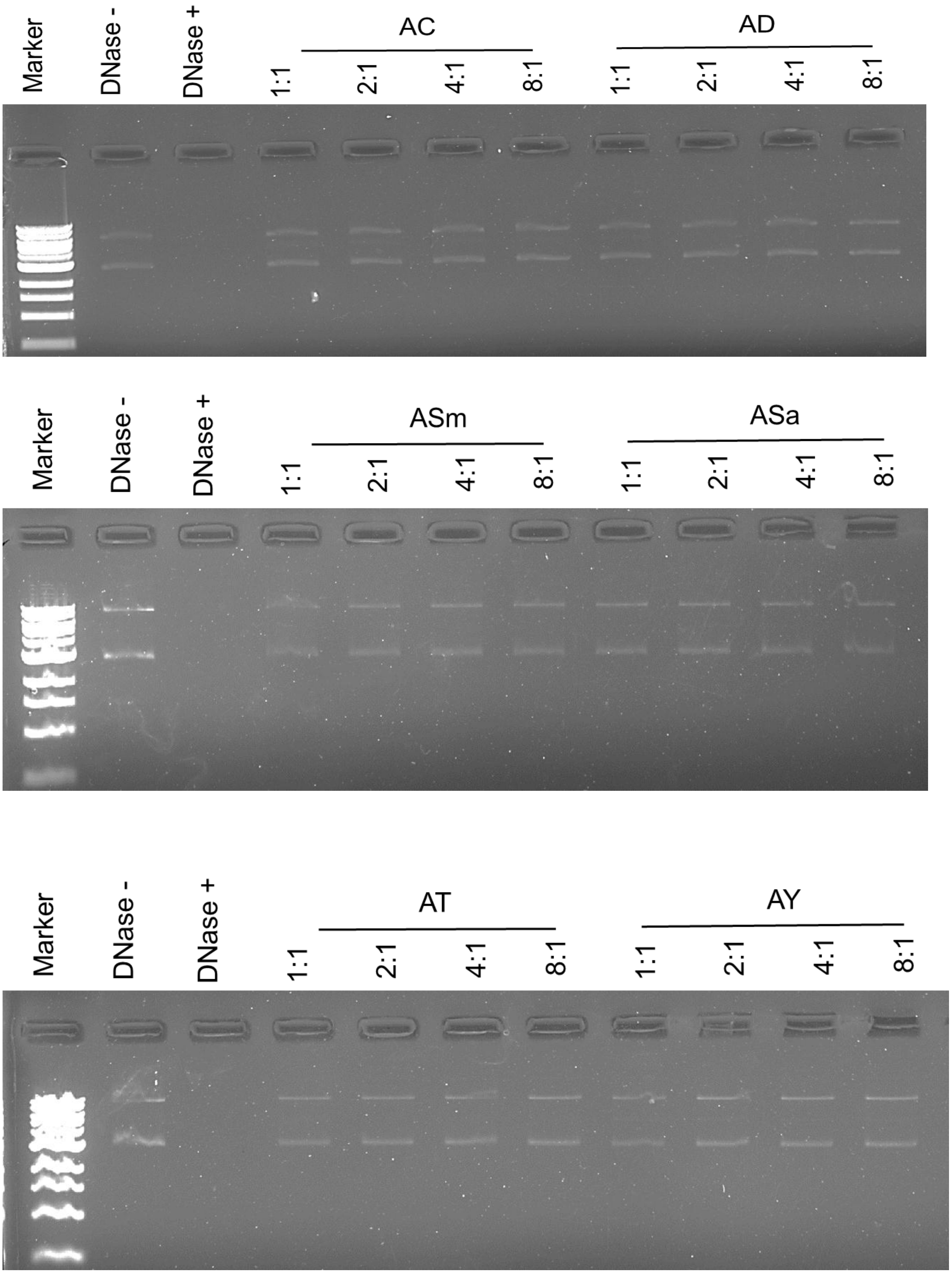

Figure S3: DNase 1 Sensitivity Assay using different sapogenin liposomes in varying charge ratios. The lipoplexes (liposome + nucleic acid) were subjected to DNase 1 enzyme followed by Proteinase K and the appearance of bands indicates the non-degradation of nucleic acid encapsulated well by liposomes.

4. Transfections in CHO and HEK293T cells

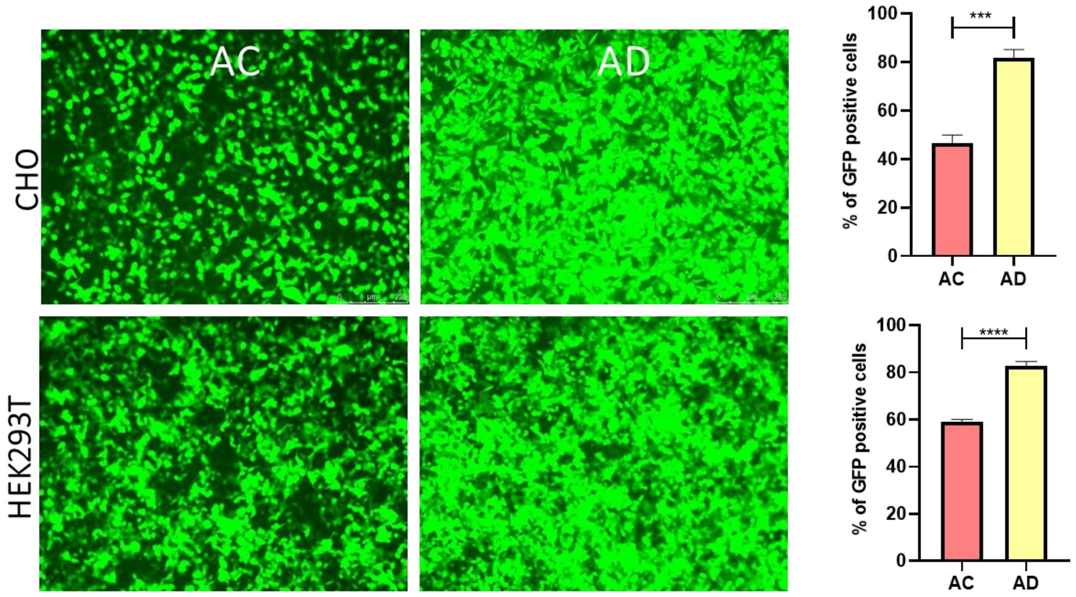

Figure S4: Transfections studies with mRNA in different cell lines using liposomes, AC & AD. Screening of AC & AD liposomes using eGFP mRNA, Representative images in epifluorescence microscope (A), Flow cytometry analysis of eGFP mRNA expression (B) in cell lines – CHO and HEK 293T.
